# Cerebrospinal fluid signs of neuronal damage after antiretroviral treatment interruption in HIV-1 infection

**DOI:** 10.1186/1742-6405-2-6

**Published:** 2005-08-18

**Authors:** Magnus Gisslén, Lars Rosengren, Lars Hagberg, Steven G Deeks, Richard W Price

**Affiliations:** 1 Department of Infectious Diseases, Göteborg University, Sahlgrenska University Hospital, Sweden; 2 Department of Neurology, Göteborg University, Sahlgrenska University Hospital, Sweden; 3 Department of Medicine, University of California San Francisco, San Francisco General Hospital, CA, USA; 4 Department of Neurology, University of California San Francisco, San Francisco General Hospital, CA, USA

## Abstract

**Background:**

The neurofilament is a major structural component of myelinated axons. Increased cerebrospinal fluid (CSF) concentrations of the light chain of the neurofilament protein (NFL) can serve as a sensitive indicator of central nervous system (CNS) injury. To assess whether interrupting antiretroviral treatment of HIV infection might have a deleterious effect on the CNS, we measured NFL levels in HIV-infected subjects interrupting therapy.

We identified subjects who had CSF HIV RNA concentrations below 50 copies/mL at the time combination antiretroviral therapy was interrupted, and for whom CSF samples were available before and after the interruption.

**Results:**

A total of 8 subjects were studied. The median (range) CSF NFL level at baseline was <125 (<125–220) ng/L (normal <250 ng/L). All 8 subjects exhibited an increase in CSF and plasma HIV RNA after stopping therapy, accompanied by intrathecal immunoactivation as evidenced by CSF lymphocytic pleocytosis (7/8 patients) and increased CSF neopterin concentration (5/6 patients). Three subjects showed a consistent increase in CSF NFL, rising from <125 ng/L to a maximum of 880 (at day 148), 1,010 (day 58) and 10,930 ng/L (day 101). None exhibited new neurological symptoms or signs, or experienced functional deterioration during the period off treatment; of 5 who underwent brief quantitative neurological testing, none showed worsening performance.

**Conclusion:**

These findings suggest that resurgence of active HIV replication may result in measurable, albeit subclinical, CNS injury. Further studies are needed to define the frequency and pathobiological importance of the increase in CSF NFL.

## Background

The mortality and morbidity of HIV infection have substantially decreased in the developed world over the past decade, largely due to the introduction of combination antiretroviral therapy (ART) [1]. Widespread use of ART has reduced nearly all of the complications of advanced HIV infection and immunosuppression in regions where therapy is available, including CNS opportunistic infections and the AIDS dementia complex (ADC) [2].

Although effective in suppressing infection, current ART regimens do not eradicate HIV, and prolonged treatment may be complicated by development of drug resistance and an array of side effects [3]. A number of therapeutic strategies have been introduced to prolong the effectiveness of antiretroviral treatment while reducing drug exposure. Although structured (or strategic) treatment interruption (STI) was initially studied as a means of enhancing HIV-specific immunity via an autovaccination phenomena or reducing the levels of drug resistant HIV, its potential therapeutic utility is now being studied primarily as a way to maintain the immunologic and clinical benefit of therapy while reducing drug-toxicity and drug-costs [4]. Despite continued concerns over the safety of interrupting therapy, a large proportion of subjects in clinical practice interrupt therapy for reasons related to drug toxicity, treatment fatigue and/or drug costs.

We undertook the current study to determine whether interrupting therapy can cause deleterious CNS effects that are not appreciated by clinical history and examination. We used archived specimens from study subjects who had interrupted therapy and been followed by repeated lumbar puncture (LP) and CSF analysis before and at various times after remaining off treatment. As a measure of neurological injury, we used concentrations of the light chain of neurofilament protein (NFL). NFL is a major structural component of myelinated axons, and the CSF level can serve as a sensitive marker of axonal damage in a number of conditions [5,6]. Both focal and systemic ischemia cause neuronal damage detectable as a leakage of NFL into the CSF proportional to the severity of the injury [5,7,8]. In chronic disorders the release of NFL is less pronounced but still raised to several times normal in active degeneration of white matter or myelinated spinal tracts, for example in multiple sclerosis and in amyotrophic lateral sclerosis [5,9,10]. Dementia of vascular etiology with subcortical white matter pathology causes moderately increased CSF NFL levels [11,12]. CSF NFL determination has thus been shown to be a versatile tool to detect neuronal damage of any cause, prompting efforts to develop alternate methods of analysis [6,13,14].

In a previous study, increased CSF NFL concentrations were found in 12 of 18 AIDS patients without CNS opportunistic infections, but in only three out of 12 subjects with less advanced, asymptomatic HIV-1 infection [15].

## Materials and methods

### Study subjects

We retrospectively identified subjects from two centers who underwent serial lumbar punctures before and after interruption of a combination antiretroviral regimen, who had undetectable HIV RNA levels in CSF at the time of the interruption and who had longitudinal samples available for our studies. All subjects participated in separate local protocols evaluating the CSF responses to changes in antiretroviral treatment. Decisions to interrupt treatment were made by the subjects and their primary caregivers independent of the CSF studies. The protocols were approved by University of California San Francisco (UCSF) Committee on Human Research (CHR) and by the Research Ethics Committee of Göteborg University. All subjects provided informed consent.

CSF viral dynamics from some of these subjects have been previously described [16,17].

### Laboratory methods

CSF concentrations of NFL were analysed using a previously described ELISA [5]. In brief, capturing antibody (hen anti-NFL IgG) was absorbed to microtest plates, and CSF samples or reference NFL were then incubated. Rabbit anti-NFL was used as secondary antibody. Bound secondary antibody was detected using peroxidase conjugated donkey anti-rabbit IgG. The sensitivity of the assay is 125 ng/L, and the upper normal reference value at the laboratory is 250 ng/L below the age of 60 years.

Cell-free CSF and plasma HIV-1 RNA were quantified with the Amplicor HIV-1 Monitor assay, version 1.5 (Roche Diagnostic System, Hoffman-La Roche, Basel, Switzerland) with a dynamic range down to 50 copies/mL and a detection limit of approximately 20 copies/mL.

Neopterin was measured by a commercially available radio-immunoassay (Henningtest Neopterin, BRAHMS, Berlin, Germany) with a normal reference value of ≤ 4.3 nmol/L in CSF [18].

Routine CSF assessments included CSF white blood cell (WBC) count and peripheral blood CD4+ T-lymphocyte (CD4) cell determination. Albumin was measured using standard Clinical Laboratory methods in each center, and the CSF:serum albumin ratios were calculated [CSF albumin (mg/L)/serum albumin (g/L)] and used as a measure of blood-brain barrier integrity [19]. The five San Francisco patients also underwent standardized neurological performance testing incorporating four tasks (timed gait, grooved pegboard with the dominant hand, finger tapping with the nondominant hand, and the Digit Symbol test of the WAIS-R), yielding an aggregate scaled z-score termed the QNPZ-4 score [20].

### Statistics

Unless otherwise indicated, descriptive group statistics in this small study are presented as median values with the range of values, and comparisons between subjects with and without increases in CSF NFL used the Mann-Whitney U test.

## Results

### Baseline characteristics

Eight subjects, five from San Francisco, California, USA and three from Göteborg, Sweden were included in the study. Demographic and baseline characteristics of the subjects are listed in Table 1. All had CSF HIV RNA concentrations <50 copies/mL and were neurologically normal except for one identified as manifesting AIDS dementia complex (ADC) Stage 0.5 (equivocal/subclinical) disease on treatment. LPs were performed at various intervals before treatment was stopped and one to six (mean 2.9) times during the period off treatment. In two subjects (207 and 223) CSF results were also available from before the initiation of treatment.

**Table 1:** Baseline characteristics of patients.

***Subject ID***	***Age***	***Sex***	***Blood CD4+***	***CDC Stage***	***ADC Stage***	***HIV-1 RNA***		***Antiretroviral therapies***
							
			***baseline (nadir)***			***CSF***	***Plasma***	
	***(years)***		***(cells/μL)***			***(log_10 _copies/ml)***		
San Francisco								
6004	56	M	275 (116)	B3	0	<1.29	4,11	d4T,3TC,IDV
6008	39	M	834 (360)	A2	0	1,61	<1.29	SQV, RTV
6011	47	M	375 (191)	B3	0,5	<1.29	1,41	ZDV, 3TC, EFV
6012	54	M	464 (199)	B3	0	<1.29	1,57	d4T, 3TC, EFV
6013	44	M	618 (275)	C2	0	<1.29	3,69	d4T, 3TC, EFV
Göteborg								
160	51	M	544 (195)	C3	0	<1.29	<1.29	ZDV, 3TC, IDV
207	47	M	428 (210)	A2	0	<1.29	<1.29	ZDV, 3TC, SQV, NFV
223	56	F	365 (110)	B3	0	<1.29	<1.29	ZDV, 3TC, IDV/RTV

### Changes in CD4-cell count, HIV RNA levels and pleocytosis

**Figure 1:**
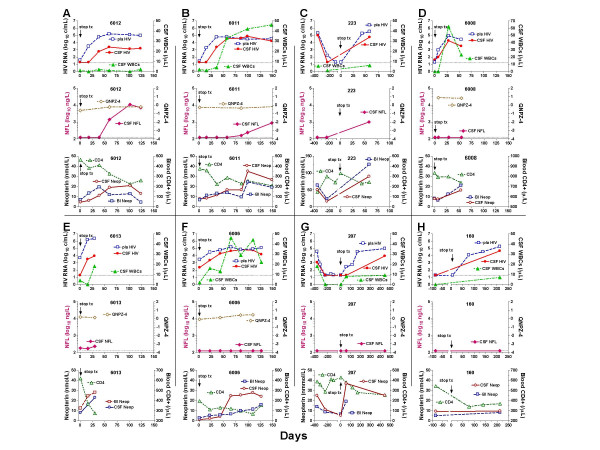
Time course of changes in salient laboratory findings in the eight subjects (A-H). Sets of three graphs for each subject arranged within eight panels (the top graph in each panel shows the changes in plasma and CSF HIV RNA and CSF WBCs, the center graph the NFL and QNPZ-4 for those with this assessment, and the bottom graph the blood and CSF neopterin and blood CD4+ T cell counts).

The time course of changes in subjects' salient laboratory findings are shown in Figure 1.

The peripheral blood CD4-cell count decreased from a median of 446 cells/μL (range, 275–834) while on treatment to 258 (208–738) cells/μL during the off-treatment follow-up.

All 8 subjects developed an increase in CSF and plasma HIV RNA concentrations. At baseline, the CSF HIV-1 RNA was <50 (<1.70 log_10_) copies/mL in all subjects, and the plasma HIV RNA levels was undetectable in six subjects. The median viral load increased to a maximum of 4.36 (3.38–4.87) log_10 _RNA copies/mL in CSF and to 5.23 (4.78–6.35) log_10 _copies/mL in plasma during the interruption period. All but one of the subjects developed an increase in CSF WBCs. CSF cell counts increased from a median of 0.5 (range, 0–4) cells/μL on treatment to a maximum of 14 (2–62) cells/μL after stopping therapy.

### NFL

Three subjects developed elevations in NFL (panels A, B and C), while the remaining five subjects did not (panels D-H). As shown in the middle panels of each triplet set, all eight subjects had normal CSF NFL concentrations (<250 ng/L) while on treatment, with six of these below the detection limit (125 ng/L). For two subjects (223 and 207, panel series C and G), CSF samples were also available before initiation of antiretroviral treatment, 9–14 months prior to baseline, and CSF NFL concentrations were found to be <125 ng/L at these intervals as well. Treatment interruption resulted in CSF NFL increases in the three subjects (6012, 6011 and 223, panels A-C), rising from baselines at the detection limits to maximum values of 880 ng/L at day 148 (6011), 10,930 ng/L at day 101 (6012) and 1,010 ng/L at day 58 (223). These increases were delayed in relation to virological changes. In subject 223 the only interval testing after treatment stopped was at day 58, while in the two others who had more frequent measurements the earliest documented increase in NFL for subject 6012 was at day 59 and for subject 6011 the inflection point showing the initial increase was delayed to 86 and 99 days.

In the two subjects with increases in NFL and more frequent measurements, the rise in plasma and CSF HIV RNA preceded that of NFL, with the plasma HIV rising by the first sampling at 17 and 15 days and the CSF by days 38 and 36. By contrast, the first change in NFL was delayed by an additional three to seven weeks. The change from baseline to maximum post-STI HIV RNA in blood and CSF was not notably different in the three subjects with increases in NFL compared to those without any change in this neural marker. There was no clear association of NFL elevation with that of CSF WBCs; indeed, the one subject without CSF pleocytosis (6012, panel A) had the greatest increase in CSF NFL.

The subjects were all clinically stable during the period of observation (median 108, range 30–446 days, after treatment interruption). None developed either AIDS-related events or neurological symptoms during the off-treatment follow-up. Likewise, their neurological examinations were unchanged, and in the five tested for quantitative neurological performance (including two with NFL elevations – subjects 6012 and 6011), none showed deterioration in their QNPZ-4 scores (shown in middle graphs of subjects' triplet panels).

### Changes in neopterin and its relationship to NFL

In keeping with the inflammatory response in CSF, all subjects in which CSF and serum neopterin were measured exhibited an increase in this marker while off therapy with the exception of subject 160 (panel H in the figure 1). CSF neopterin rose from a median of 6.65 (range, 1.80–16.4) to 23.0 (6.30–90.0) nmol/L in the six subjects analysed. Where the timing could be evaluated, the peak increase in CSF neopterin appeared to be delayed compared to that of CSF HIV, and in subjects 6012 and 6011 occurred near the time of NFL rise. However, based on these limited data, it was not clear that increases in CSF neopterin correlated with increases in NFL. For example, the increase in CSF neopterin in subject 207 (panel G) exceeded that of subjects 6012 and 6011 despite continued undetectable CSF NFL.

The CD4-cell decreases began earlier than the increases in NFL in subjects 6012 and 6011. Again, there was no clear association of development of NFL increase with baseline or nadir CD4 counts or the magnitude of their cell decline. Also, only one subject (223) showed evidence of blood-brain barrier disruption with an elevated CSF:serum albumin ratio. In all others the albumin ratios were stable or showed only minor change that did not parallel changes in CSF NFL (data not shown). As expected within this small dataset, no statistical significant difference could be found between subjects with or without CSF NFL increase, neither regarding the magnitude of increase in CSF neopterin, CSF WBC, CSF nor plasma viral load (data not shown).

## Discussion

While the use of STI to stimulate and boost the immune system by re-exposure to viral antigens has not been proved effective and may be complicated by enduring T cell loss and enhanced resistance [4,21], there is still interest in judicious suspension of therapy in order to reduce cost and, more particularly, side effects of therapy. Additionally, patients may stop treatment for personal reasons or because of toxicities. Our observation of increased CSF NFL in three of eight subjects raises a previously unexamined potential concern when interrupting antiretroviral treatment – the possibility of nervous system injury – although further studies are needed to confirm the frequency of this finding and, more particularly, to understand its pathobiological and clinical implications.

The neurofilament is a major structural element of neurons, found most conspicuously in larger neurons and their myelinated axons. It is composed of a triplet protein of which the light subunit (NFL) is an essential component of the neurofilament core. Its main function is to maintain the axonal calibre and it thereby has a crucial role in the structural and functional integrity of axons and in their capacity to rapidly conduct nerve impulses [22]. Increased CSF NFL concentrations are thought to reflect principally injury of myelinated axons, and a clear association has been found between the presence of white matter changes and increased CSF NFL levels in patients with Alzheimer's disease and subcortical vascular dementia [12]. CSF NFL is also increased in several other neurodegenerative disorders linked to demyelination and/or axonal degeneration, including active multiple sclerosis and amyotrophic lateral sclerosis [5,10].

CSF NFL increased substantially in three of our eight subjects studied after stopping antiretroviral therapy, thereby indicating brain injury in this setting. The mechanisms responsible for this rise in NFL remain to be determined. Indeed, from a virological perspective, it is uncertain whether the increase in NFL is simply a complication of established viremia or is related more particularly to the abrupt surge in viral replication that follows treatment interruption. In other words, was this neurological damage due simply to higher levels of HIV replication or due to the "shock" associated with a rapid resurgence after interruption? Perhaps favouring the latter is the finding of elevations in NFL in these subjects without an AIDS diagnosis and CD4 counts above 200 cells/μL, contrasting somewhat with our earlier study that showing CSF NFL elevations chiefly in subjects with AIDS or with CNS opportunistic infections [15]. Normal CSF NFL concentration in one patient measured before treatment initiation gives further support to a rapid viral rebound as the triggering event.

As seen by others [23], plasma viral load increased rapidly and was already detected at the first follow-up (median 18 days) after treatment interruption. Subsequently, CSF viral load increased after a short interval in the subjects with sufficient observations to define these temporal relationships. In a larger experience, that included some of these same subjects, we have noted this delay and also found that an increase in CSF lymphocytosis developed in more than half of subjects [16,17].

The current study also shows that STI leads to increases in CSF and plasma neopterin levels and that these elevations peak later than plasma and CSF HIV RNA levels. Neopterin is an unspecific marker of immune activation that is largely derived from activated macrophages and microglia [24]. CSF neopterin is increased in patients with ADC and has also been found to be a predictive marker of ADC [25]. However, CSF neopterin is also frequently increased in HIV-infected patients without neurological complications, with higher levels found in severely immunocompromised patients than in those with CD4-cell counts above 200 cells/μL [26]. In a previous study, we found an association between increased NFL and CSF neopterin concentrations [15]. This led to our suggesting that immune activation was important in the neuronal injury and release of NFL in this setting. While elevations in CSF neopterin were found in all the subjects with increased NFL and there was the suggestion of temporal association, we also observed subjects with elevated CSF neopterin and normal, unchanging CSF NFL. Hence, further studies are needed to examine this association.

Whatever the underlying mechanism, the presumed axonal damage was subclinical, and no concomitant neurological deterioration was detected during the follow-up period either on the basis of symptoms or on clinical or more formal, though brief, quantitative testing. Based on this initial study, it remains unknown whether this axonal injury has clinical importance. The subject number was small and the follow-up period, for the most part, of limited duration. Hence, longer-term impact on neurological function could not be discerned, though one subject was followed for two years off treatment and did not exhibit neurological change over that time. With one exception (this same subject, 6011, with ADC 0.5 [27]), our subjects were neurologically normal and without prior evidence of ADC. Theoretically, for patients with ADC, resurgence of viremia and CNS infection might be more hazardous, and stopping treatment should be undertaken with particular caution. Whether treatment approaches with repeated cycles of treatment interruptions also can initiate repeated damage to the brain is an open question.

The present study also does not establish whether axonal injury is an acute short-term event after treatment interruption or if it is more chronic. In herpes simplex type 1 (HSV-1) encephalitis, CSF NFL levels increase to a maximum approximately 8–14 days after the onset of neurological symptoms, and only slowly decrease thereafter, with abnormal levels still detected as long as 3–10 months later [28]. Similar observations, with slow normalization of CSF NFL concentrations, have also been made after focal brain ischemia and after acute relapses of multiple sclerosis [10]. Wallerian degeneration, with an anterograde degeneration of axons and disruption of the axonal cytoskeleton, has been suggested as a principal cause of this delayed and long-lasting CSF NFL increase in these settings. The metabolic degradation and half-life of NFL in CSF is also not established.

The temporal delay between viral replication (as evidenced by plasma and more particularly CSF HIV RNA levels) and the onset of axonal disturbance (measured by NFL) may also imply that Wallerian degeneration is involved in STI. If the neuronal soma is the primary site of action, the axonal changes should be a later event. A direct effect on axons in myelinated tracts or spinal roots could also be hypothesised. Alternatively, there may be a lag between HIV replication and onset of neural injury, perhaps involving immunopathological pathways that lag behind the surge in viral replication. The magnitude of the CSF viral load increase, however, did not directly predict the development of elevated CSF NFL; as noted earlier, there were subjects with high CSF viral load without change in NFL levels.

## Conclusion

In conclusion, our study raises the question of whether treatment interruption enhances the risk of brain injury. Conversely, it may also suggest that once started, continued treatment might prevent subclinical brain damage. NFL is a sensitive marker of axonal injury which in the present setting seems to disclose subclinical injury. Further studies are needed to define the frequency, duration, magnitude and pathobiological importance of this increase in CSF NFL in HIV infection.

## Competing interests

The author(s) declare that they have no competing interests.

## Authors' contributions

MG, LR, LH, and RWP contributed to the conception of the study, data interpretation, and writing of the paper. SGD contributed to the establishment of the clinical study and writing the paper.
